# Supplementary material for: Improving heat and mass transfer rates through continuous drop-wise condensation
Source: Sci Rep. 2021 Oct 4;11:19636. doi: 10.1038/s41598-021-98992-9 (PMC8490593; doi:10.1038/s41598-021-98992-9)
Supplement: Supplementary file 7 — Supplementary Information 7. [file 41598_2021_98992_MOESM7_ESM.pdf]

Supplementary Material: Improving Heat and Mass Transfer Rates Through Continuous Drop-Wise Condensation

Ali Alshehri,[1,2*] **Jonathan P. Rothstein,**[3] **H. Pirouz Kavehpour**[1]

[1]Mechanical and Aerospace Engineering Department, University of California, Los Angeles, CA 90095, USA,
[2]Mechanical Engineering Department, King Fahd University of Petroleum and Minerals, Dhahran 31261, Saudi Arabia,
[3]Department of Mechanical and Industrial Engineering, University of Massachusetts Amherst, Amherst, Massachusetts 01003-2210, USA.
*To whom correspondence should be addressed; E-mail: aalshehri@ucla.edu.

ABSTRACT

S-1 Experimental setup

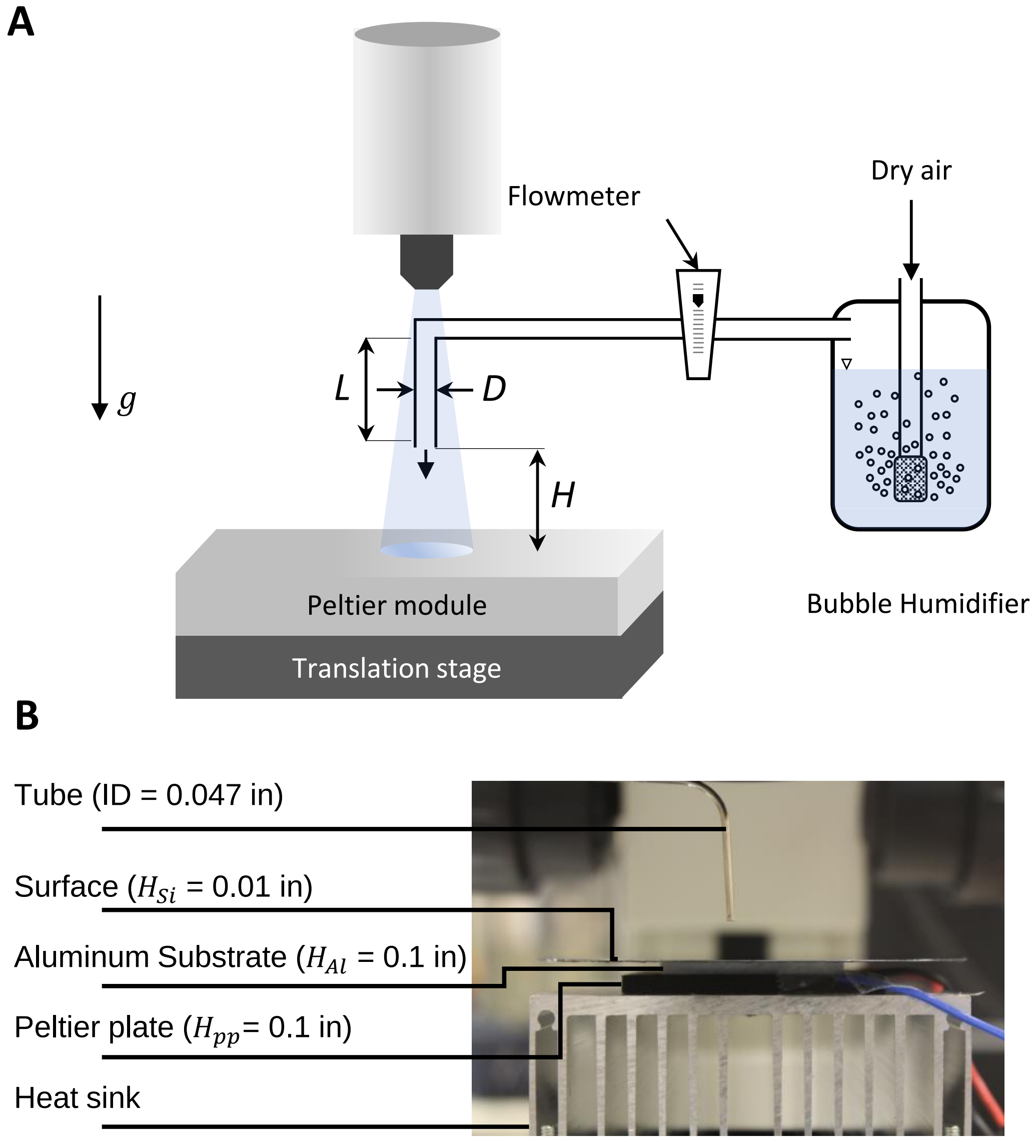

Figure S1. Continuous Drop-wise Condensation experimental setup. A. A schematic of CDC illustrating the general setup for condensation experiments. Dry air is bubbled into a room-temperature pool of DI water through several spargers (one is shown for illustration). The different parameters are discussed in the method section. **B.** A side view of the condensation surface assembly.

S-2 Surface characterization

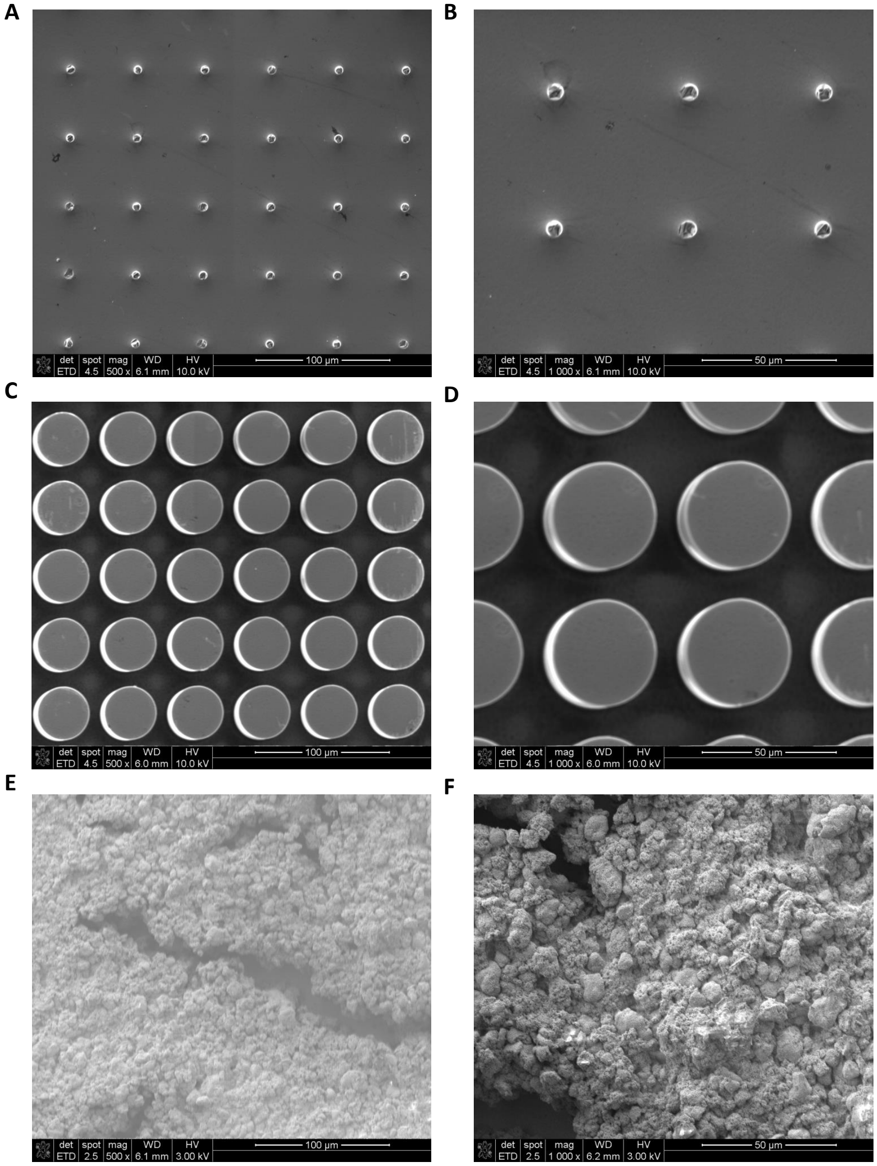

Figure S2. SEM images of the condensation surfaces. **A & B.** Two magnifications of the Hydrophilic Microstructured surface (θ_A = 70° and θ_R = 50°). **C & D.** Two magnifications of the Superhydrophobic Microstructured surface (θ_A = 160° and θ_R = 127°). **E & F.** Two magnifications of the Superhydrophobic Nanostructured surface (θ_A = 157° and θ_R = 154°).

S-3 Heat Transfer of water Vapor Condensation with Humid Air Jet Impingement

In this section, we present the procedure of evaluating the overall heat transfer coefficient due to dehumidification. It has been shown that the existence of minute amounts of non-condensable gases (NCG), such as air reduces the condensation rate, and thus the heat flux, tremendously. This is due to the fact that at steady state conditions, a diffusion layer builds on top of the condensate formed on the surface. The condensation of vapor becomes mainly dominated by the mass diffusion through this layer. The thermal resistance of this layer dominates the heat transfer to the surface. However, without delving into modeling the dehumidification process, here we evaluate experimentally the overall heat transfer coefficient.

Initially, we measured the growth of condensate droplets optically using several microscopic images at different elapsed times. In Fig.S3, we present the observed condensed mass per unit surface area vs. elapsed time at varying jet Reynolds number for the case of hydrophobic surface $\theta_A/\theta_R = 107^o/103^o$. The condensate mass was evaluated as indicated in Eq.4 (main text). It is noted that the condensed mass flux becomes eventually linear with elapsed time indicating a steady state condensation process. The slope of this line is a representative estimation of the condensation rate per unit surface area. It is worth mentioning that a circular area with a diameter of 1 mm was chosen as the condensation area, which lies within the stagnation region of the impinged jet. The mass transfer coefficient is then obtained using Eq.3 (main text).

The heat transfer flux to the condensation surface can be estimated by the following relation.

$$q''(r) = U(T_\infty - T_s) = \dot{m}''h_{fg} + h(T_\infty - T_s) \tag{S-3.1}$$

where U is the overall heat transfer coefficient, h_{fg} is the latent heat of vaporization, h is the convective heat transfer coefficient of the gas-vapor side, T_∞ and T_s are the ambient and surface temperatures, respectively. Typically, the latent heat transfer is much greater than the sensible heat, therefore, we only consider the latent heat part in Eq. S-4.1. In Fig.4A (main text), we plot the experimental evaluation of the heat and mass transfer coefficients at varying jet Reynolds numbers.

For comparison of the current method of dehumidification with literature dehumidifiers, we think CDC provides an extremely compact condenser design. Therefore, we compare the different state-of-the-art dehumidifiers by a compactness factor which is given by the following relation[1].

$$C_h = \frac{UA}{V} \tag{S-3.2}$$

$$C_m = \frac{h_m A}{V} \tag{S-3.3}$$

where C_h and C_m are the compactness factor of heat and mass transfer exchangers, respectively. The higher the value indicates a higher transfer rate per unit driving potential (temperature or vapor mass fraction) per unit volume. In Fig.4B, we show a comparison of the compactness factor for different dehumidifiers along with the current method. We reconstruct the results published by Sadeghpour et al.[1]. Table S1 summarizes the parameters used for the comparison.

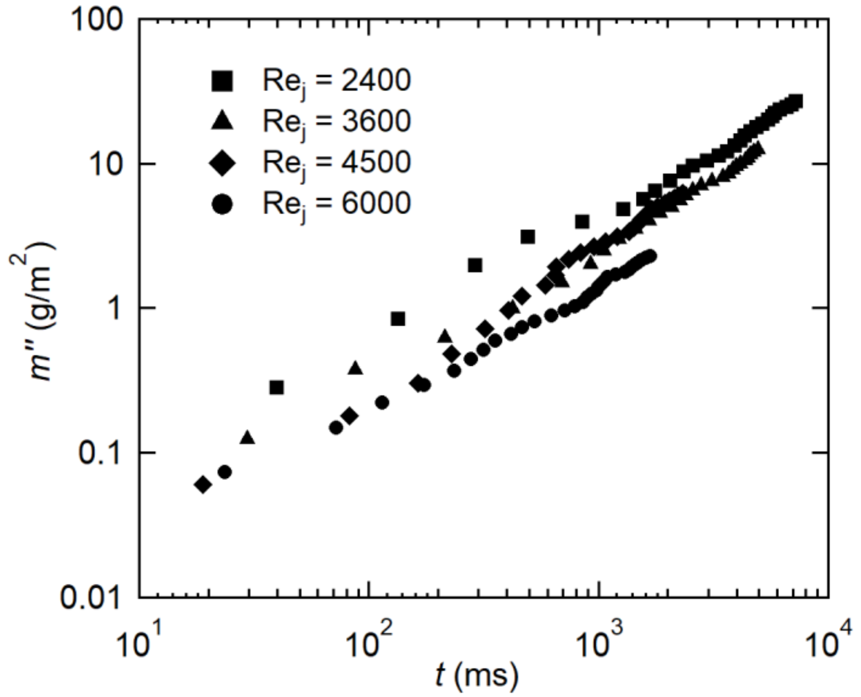

Figure S3. Condensation mass flux as a function of elapsed time for different jet Reynolds numbers for the case of hydrophobic surface $\theta_A/\theta_R = 107^o/103^o$.

Table S1. Summary of the parameters used to compare state-of-the-art dehumidifiers.

Dehumidifier type	A/V (m^{-1})	h_m (kg/m^2-s)	C_m (kg/m^3-s)	q'' (kW/m^2)	ΔT (^{o}C)	U (W/m^2-K)	C_h (kW/m^3-K)
Flat plate	108	0.0148	1.6	3.7	19.8	187	20.3
Bubble Column	16	0.081	1.3	15	28	536	8.7
Moving Liquid beads	62	0.072	4.5	2.1	7.3	288	17.9
CDC (current method)							
Re_j = 1200	67	3.6	241.2	44.2	6	7362	4908
Re_j = 2400	67	0.75	50.2	9.2	6	1533	1022
Re_j = 3600	67	0.5	33.8	6.2	6	1031	687
Re_j = 4500	67	0.57	37.9	6.9	6	1156	770
Re_j = 6000	67	0.27	18.3	3.4	6	563	376

S-4 Heat Transfer of Pure Vapor with Jet Impingement

In this section, an estimate of the heat transfer of DWC of pure vapor is presented. We utilize the theoretical model developed originally by Rose and co-workers[2]. The process of DWC can be summarized by the following sequence of events; initial nucleation of vapor clusters, droplet growth by condensation on the interface, droplet growth by coalescence, droplet fall off, and finally re-nucleation of droplets.

The first process of DWC is heterogeneous condensation over active nucleation sites on the condensation substrate. The active nucleation site density (N_s) depends on surface topography, Fluid's thermophysical properties, and subcooling degree[3,4]. The value of active nucleation site density is in the range of 10^9-10^{15} m^{-2}[5]. For the purpose of obtaining an approximate comparison between jet- and gravity-assisted shedding mechanisms, we choose a value for $N_s = 10^{12}$ m^{-2}. The smallest stable droplet formed by condensation in the nucleation site is given as[5,6]

$$r_{min} = \frac{2T_{sat}\gamma}{\rho_l h_{fg}(T_{sat} - T_s)} \tag{S-4.1}$$

where T_{sat}, γ, ρ_l, h_{fg}, T_s as saturation temperature, surface tension, liquid density, latent heat of vaporization, and surface tension, respectively. It has been shown experimentally that droplets in the range $[r_{min}, r_e]$ grow by direct condensation only, where the effective radius results from geometrical argument as $r_e = 1/\sqrt{4N_s}$. Droplets with radii higher than the effective radius grow by direct condensation on their surfaces as well as by coalescence with neighboring droplets. DWC on vertical surface, i.e. typical configuration, are characterized by the existence of a maximum droplet radius of which droplets start sliding on the surface and consequently sweeping smaller droplets in their path. A balance between the weight of the droplet and the surface retention force results in the following relation of maximum radius for gravity-assisted shedding[7]

$$r_{max,g} = \sqrt{\frac{6\gamma \sin\theta(\cos\theta_r - \cos\theta_a)}{\pi\rho_l g(2 - 3\cos\theta + \cos^3\theta)}} \tag{S-4.2}$$

where θ, θ_r, θ_a are the static, receding and advancing contact angles respectively and g is the gravitational acceleration. To estimate the overall heat transfer rate to the condensation surface, several researchers have utilized the theory developed by Rose and co-workers[2]. The model is centered around correlating the heat transfer across a single droplet to the overall heat transfer across the entire droplets on a surface. The following formula is usually considered for the overall heat transfer rate

$$q'' = \int_{r_{min}}^{r_e} q_d(r,\theta)n(r,\theta)dr + \int_{r_e}^{r_{max}} q_d(r,\theta)N(r,\theta)dr \tag{S-4.3}$$

where $q_d(r,\theta)$, $n(r,\theta)$ and $N(r,\theta)$ are the heat transfer through a single droplet of radius (r), the *small* droplet size

distribution in the range $[r_{min}, r_e]$, and *large* droplet size distribution in the range $[r_e, r_{max}]$, respectively. The detailed derivation of the individual parameters has been discussed in several papers beginning with the work of Rose[2].

The heat transfer across a single droplet of radius (r) can be represented as a combination of Laplace pressure effect due to curvature, liquid-vapor interfacial thermal resistance (Knudsen layer), conduction through the droplet body, and conduction through the condensation surface. heat transfer across a single droplet can be written as[8]

$$q_d(r,\theta) = \pi r^2 (T_{sat} - T_s - \frac{2T_{sat}\gamma}{\rho_l h_{fg} r})(\frac{1}{2h_i(1-\cos\theta)} + \frac{r\theta}{4k_l \sin\theta} + \frac{\delta_s}{k_s \sin^2\theta})^{-1} \tag{S-4.4}$$

where k_l and k_s are the thermal conductivity of the liquid and the condensation surface, respectively and δ_s is the thickness of the condensation surface. The liquid-vapor interfacial heat transfer coefficient is given as[9]

$$h_i = \frac{2\sigma_c}{2-\sigma_c}\sqrt{\frac{M}{2\pi R T_s}}\frac{h_{fg}^2 \rho_v}{T_s} \tag{S-4.5}$$

where σ_c, M, R and ρ_v are the condensation coefficient, molecular weight of water, gas constant, and water vapor density, respectively. The size distribution of large droplets was derived experimentally and mathematically as[10,11]

$$N(r,\theta) = \frac{1}{3\pi r_{max} r^2}\left(\frac{r}{r_{max}}\right)^{-2/3} \tag{S-4.6}$$

Lastly, the population balance theory was used to derive the small droplet size distribution[12]. The form is given as

$$n(r,\theta) = \frac{1}{3\pi r_{max} r_e^3}\left(\frac{r_e}{r_{max}}\right)^{-2/3} \frac{r(r_e - r_{min})}{r - r_{min}} \frac{A_2 r + A_3}{A_2 r_e + A_3} \exp(B_1 + B_2) \tag{S-4.7}$$

where the constants are given as

$$A_1 = \frac{(T_{sat} - T_s)}{2\rho_l h_{fg}}, \tag{S-4.8a}$$

$$A_2 = \frac{\theta(1-\cos\theta)}{4k_l \sin\theta}, \tag{S-4.8b}$$

$$A_3 = \frac{1}{2h_i} + \frac{\delta_s(1-\cos\theta)}{k_s \sin^2\theta} \tag{S-4.8c}$$

$$A_4 = \frac{A_2}{\tau A_1}\left[\frac{r_e^2 - r^2}{2} + r_{min}(r_e - r) + r_{min}^2 \ln\left(\frac{r_e - r_{min}}{r - r_{min}}\right)\right] \tag{S-4.8d}$$

$$A_5 = \frac{A_3}{\tau A_1}\left[r_e - r + r_{min}\ln\left(\frac{r_e - r_{min}}{r - r_{min}}\right)\right] \tag{S-4.8e}$$

$$\tau = \frac{3r_e^2(A_2 r_e + A_3)^2}{A_1[8A_3 r_e - 14A_2 r_e r_{min} + 11A_2 r_e^2 - 11A_3 r_{min}]} \tag{S-4.8f}$$

Equations S-4.1 through S-4.8 along with known thermophysical properties provide a complete set of equations to obtain the overall heat transfer rate to a surface. For instance, Fig.S4 shows the heat flux variation on a surface with different wettability,

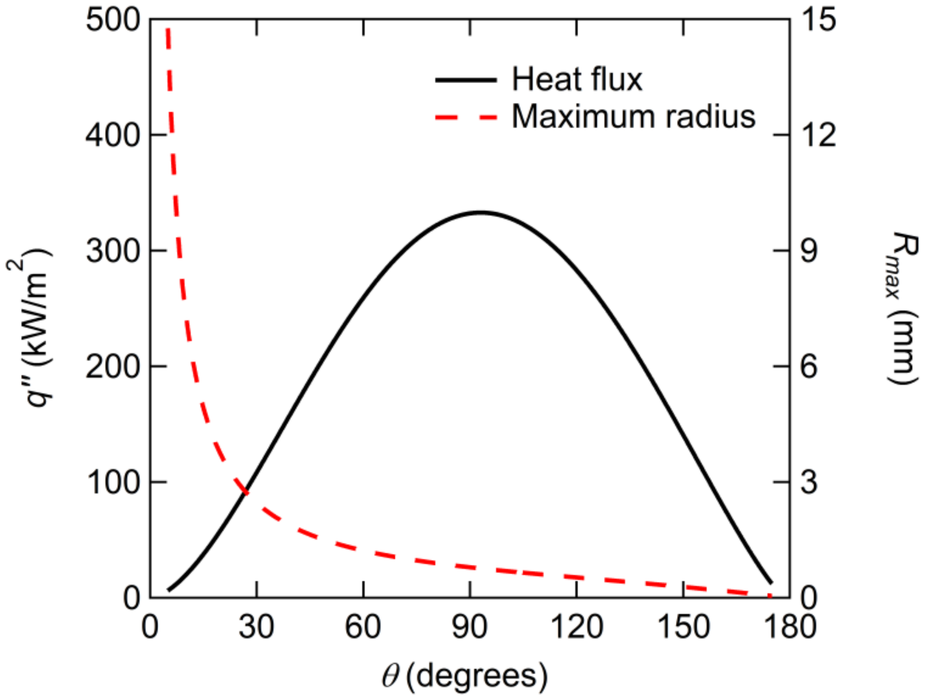

Figure S4. Heat flux to a vertical surface exposed to pure vapor analytically evaluated at different static contact angles with gravity-assisted shedding. The parameters inputted into the model are; T_{sat}=22°C, T_s=15°C, δ_s = 254μm, k_s=100W/m^{2}K, N_s=10^{12} sites/m^2, σ_c=1, and $\theta_A - \theta_R$=5°.

i.e. contact angles, under gravity-assisted droplet shedding. In the figure, we also show the maximum radius of droplets being shed by the assistance of body weight. For a surface with static contact angle of 90°, we notice that a heat flux of 300 kW/m^2 could be transferred to the surface with a maximum droplet radius of 790μm. With higher contact angle surfaces, droplets of lower radius could be shed. However, due to the increased conduction resistance, heat flux drops for extremely non-wettable surfaces.

In Fig.S5, we show for a constant static contact angle, the effect of varying the maximum droplet radius. The cross symbols in the figure represent the case of gravity-assisted droplet shedding as their respective static contact angles. For instance, the heat flux to a surface could be enhanced by 150% if a coating with static contact angle of 160° is used with a maximum droplet radius of around 20 micron compared with the hydrophilic surface and gravity-assisted case.

We believe that with CDC, the mechanism of droplet growth would be similar to that of regular drop-wise condensation with the maximum droplet radius determined by the jet impingement shedding action. Therefore, in Fig.4D we normalize the heat flux and maximum droplet radius with with their respective values due to gravity-assisted drop-wise condensation.

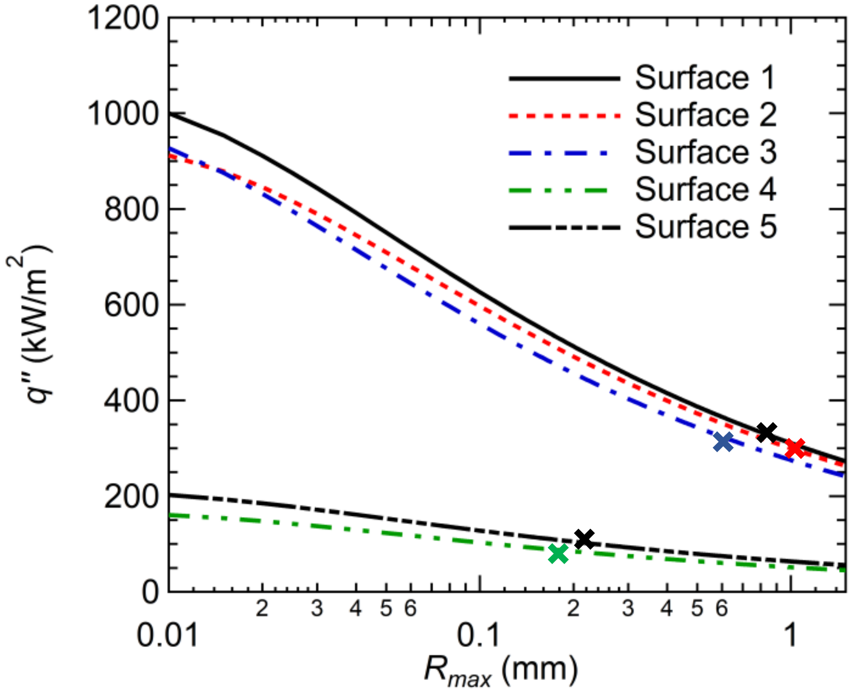

Figure S5. Heat flux to a vertical surface exposed to pure vapor analytically evaluated at maximum droplet Radii for the different tested surfaces. The cross symbol represents the value obtained with gravity-assisted shedding. The parameters inputted into the model are similar to that in Fig.S4 for meaningful comparison.

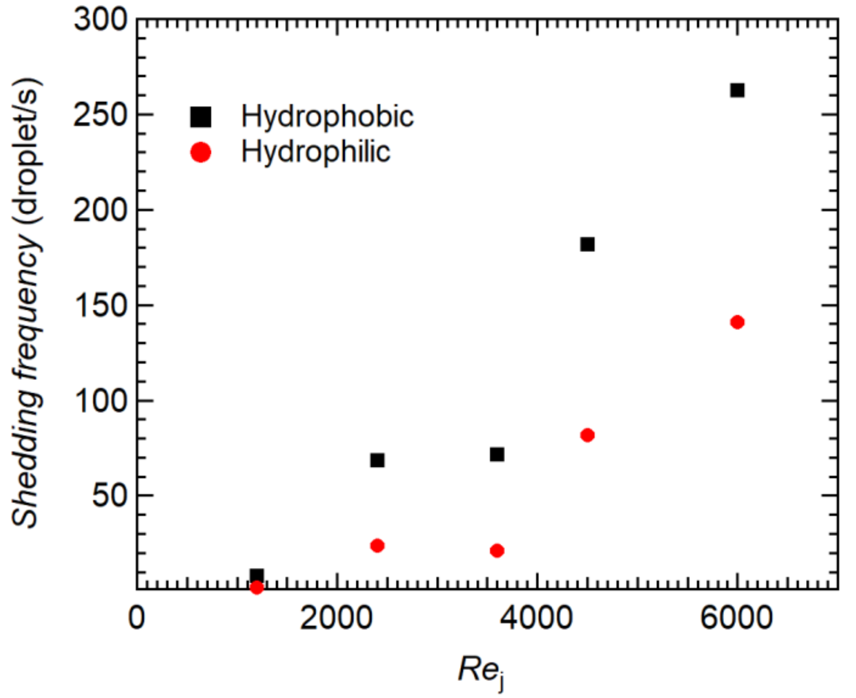

Figure S6. Improved droplet shedding frequency under different jet Reynolds numbers (Re_j) for two different surfaces θ_A/θ_R = $107^o/103^o$ and $\theta_A/\theta_R = 85 \pm 2^o/72 \pm 2^o$.

S-5 Discussion of drag force quantification

As observed in (Fig.1 and Fig.2 of main manuscript), the initial droplet shedding occurs within the stagnation region, i.e. almost within a tube radius from the stagnation point. It is well established that for the case of laminar non-mixing gas jet impingement, the similarity solution of Navier-Stokes equations gives a constant momentum boundary layer thickness in the impingement region. The equation of the boundary layer thickness is usually written as $\delta_o \sim \sqrt{\nu D/u_j}$, where ν is the kinematic viscosity of the jet, D is the diameter of the tube exit, and u_j is the speed of the jet at the standoff distance[13,14]. In this work, the jet Reynolds number was in the range of 1000-6000, therefore, the momentum boundary layer thickness should be below the range of 15 - 35 μm, respectively. Even though these estimations are for laminar jets ($Re < 1000$), turbulent mixing, like in our experiments, results in a lower boundary layer thickness. Our observations of droplets size show that the boundary layer thickness is smaller than the smallest droplet being shed. Therefore, we expect that the flow field within the boundary layer is unimportant.

In general, the drag force on the droplet due to the jet flow can be given by equation 7. The projected area shape factor Γ_A can be obtained geometrically as

$$\Gamma_A = \theta - \frac{1}{2}\sin 2\theta \qquad \text{(S-5.1)}$$

The drag coefficient based on the jet speed and droplet diameter is in the range of 1-0.6 for Reynolds numbers of 100-500, respectively. These values were obtained from spherical relations for the lack of better quantification in the literature, however it is a common practice. The effective velocity term is taken to equal the jet mean velocity value multiplied by a proportionality constant, i.e. $u_o = av_j = Q/A_{tube}$.

S-6 Droplet equation of motion: simplistic approach

In this section, we present a simplistic equation of motion that represents a one-dimensional force balance on a single droplet. In Fig.S7A, we show the simplified physical model of a droplet with diameter (D) and contact angle (θ) located at a distance (x_o) from the center of impingement region. The jet issues from a tube that is located at a standoff distance (H) with a mean velocity (v_j). The only forces responsible for droplet movement are drag force (F_d), surface tension force (F_s), and viscous friction force (F_v) (Fig.S7B). Newtons seconds law is applied to the droplet as follows.

$$\rho_l \Gamma_v \frac{d(D^3 v)}{dt} = \frac{1}{8} C_d \rho_g \Gamma_A (u_o - v)^2 D^2 - \Gamma_L D \gamma (\cos\theta_R - \cos\theta_A) - \eta \Gamma_{base} R^2 \frac{\partial v}{\partial y}\Big|_{base} \tag{S-6.1}$$

where ρ_l is the density of condensate liquid, v is the local velocity of the droplet, Γ_v is the volumetric shape factor, η is the dynamic viscosity, Γ_{base} is the shape factor of the base area of the drop. At the onset of droplet motion, only the drag and surface tension forces are present. Equation 8 in the main manuscript is the resultant of the force balance. In the accelerating droplet region (Period II), the drag force becomes significantly higher than the retention forces. Hence, Eq.S-6.1 is reduced to the following.

$$\rho_l \Gamma_v \frac{d(D^3 v)}{dt} = \frac{1}{8} C_d \rho_g \Gamma_A (u_o)^2 D^2 \tag{S-6.2}$$

This equation suggests a negligible effect of surface wettability on droplet motion in regions corresponding to Period II. This is clear from the droplet apparent velocity shown in Fig.6D. In the decelerating period (Period III), Eq.S-6.1 should be fully used. However, because of the complexity of determining the coefficients in the equation, presenting an outline of the equation is sufficient.

A

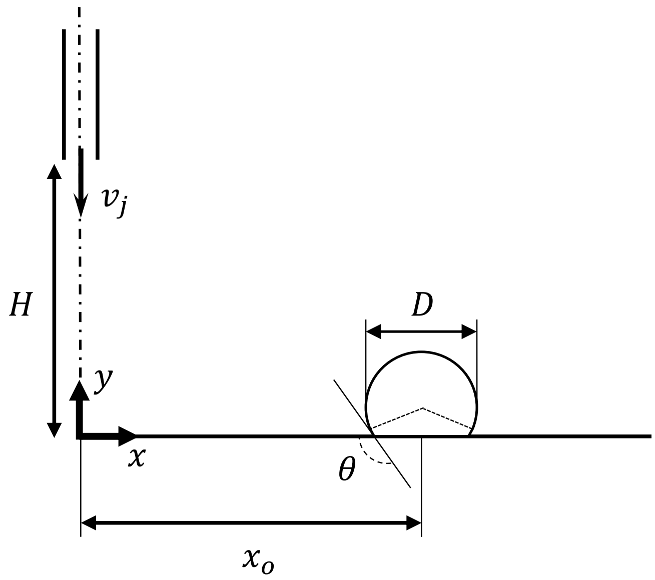

B

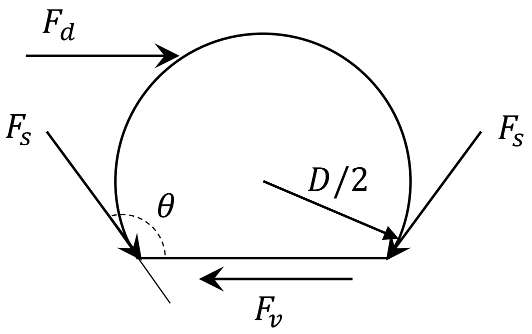

Figure S7. Physical model for writing the equation of motion of a single droplet in contact with flow of an axisymmetric jet. **A.** one-dimensional schematics of the pertaining parameters. **B.** Forces acting on a single droplet under a generalized case of a moving droplet.

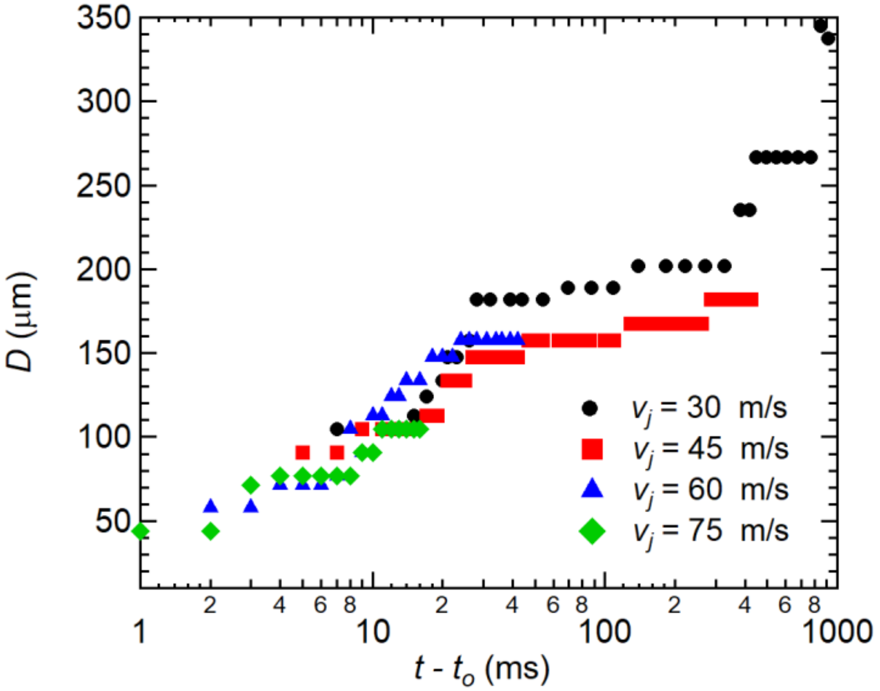

Figure S8. Single droplet growth as it departs the impingement region at various jet Reynolds numbers on the hydrophobic surface. The values correspond to similar conditions of Fig.7A

S-7 Videos

Video S1 10-second Condensation process on surface 1 (hydrophilic Silicon surface) at different jet Reynolds numbers. The experimental conditions are as indicated in the method section while surface characteristics are as in Table S1.

Video S2 10-second Condensation process on surface 2 (hydrophilic silicon surface with high contact angle hysteresis) at different jet Reynolds numbers. The experimental conditions are as indicated in the method section while surface characteristics are as in Table S1.

Video S3 10-second Condensation process on surface 3 (hydrophobic Silicon surface) at different jet Reynolds numbers. The experimental conditions are as indicated in the method section while surface characteristics are as in Table S1.

Video S4 10-second Condensation process on surface 4 (superhydrophobic silicon surface with high contact angle hysteresis) at different jet Reynolds numbers. The experimental conditions are as indicated in the method section while surface characteristics are as in Table S1.

Video S5 10-second Condensation process on surface 5 (superhydrophobic silicon surface with negligible contact angle hysteresis) at different jet Reynolds numbers. The experimental conditions are as indicated in the method section while surface characteristics are as in Table S1.

Video S6 Illustration of droplet jumping on surface 5 (superhydrophobic silicon surface with negligible contact angle hysteresis).

References

1. Sadeghpour, A. *et al.* Water vapor capturing using an array of traveling liquid beads for desalination and water treatment. *Sci. advances* **5**, eaav7662 (2019).
2. Rose, J. W. Personal reflections on fifty years of condensation heat transfer research. *J. Enhanced Heat Transf.* **22** (2015).
3. Rose, J. Further aspects of dropwise condensation theory. *Int. J. Heat Mass Transf.* **19**, 1363–1370 (1976).
4. Sikarwar, B. S., Khandekar, S. & Muralidhar, K. Mathematical modelling of dropwise condensation on textured surfaces. *Sadhana* **38**, 1135–1171 (2013).
5. Liu, X. & Cheng, P. Dropwise condensation theory revisited part ii. droplet nucleation density and condensation heat flux. *Int. J. Heat Mass Transf.* **83**, 842–849 (2015).

6. Graham, C. & Griffith, P. Drop size distributions and heat transfer in dropwise condensation. *Int. J. Heat Mass Transf.* **16**, 337–346 (1973).

7. Dimitrakopoulos, P. & Higdon, J. On the gravitational displacement of three-dimensional fluid droplets from inclined solid surfaces. *J. Fluid Mech.* **395**, 181–209 (1999).

8. Kim, S. & Kim, K. J. Dropwise condensation modeling suitable for superhydrophobic surfaces. *J. heat transfer* **133** (2011).

9. Wen, H. W. & Jer, R. M. On the heat transfer in dropwise condensation. *The chemical engineering journal* **12**, 225–231 (1976).

10. Le Fevre, E. & Rose, J. W. A theory of heat transfer by dropwise condensation. In *International Heat Transfer Conference Digital Library* (Begel House Inc., 1966).

11. Rose, J. & Glicksman, L. Dropwise condensation—the distribution of drop sizes. *Int. J. Heat Mass Transf.* **16**, 411–425 (1973).

12. Abu-Orabi, M. Modeling of heat transfer in dropwise condensation. *Int. journal heat mass transfer* **41**, 81–87 (1998).

13. Schlichting, H. Boundary layer theory. hcgraw-hill book company. *New York, N. Y* 586 (1968).

14. Lienhard, J. Liquid jet impingement. *Annu. Rev. Heat Transf.* **6** (1995).
